# Choroidal–ventricular system abnormalities are linked to amyloid‐*β* aggregation in Alzheimer's disease

**DOI:** 10.1002/alz.71205

**Published:** 2026-02-25

**Authors:** Seyyed Ali Hosseini, Etienne Aumont, Nesrine Rahmouni, Marcel S Woo, Arthur C. Macedo, Brandon Hall, Tevy Chan, Joseph Therriault, Lydia Trudel, Yansheng Zheng, Gleb Bezgin, Aurélie Lebrun, Jaime Fernandez Arias, Kely Quispialaya Socualaya, Cécile Tissot, Stijn Servaes, Yi‐Ting Wang, Delphine Oliva‐Lopez, Stuart Mitchell, Robert Hopewell, Chris Hung‐Hsin Hsiao, Catherine Saleh, Jenna Stevenson, Firoza Lussier, Liyong Wu, Min Chu, Sanjeev Chawla, Vladimir Fonov, Gassan Massarweh, Yasser Iturria Medina, Jean‐Paul Soucy, Karine Provost, David A. Rudko, Thomas Karikari, Andréa Lessa Benedet, Nicholas J. Ashton, Henrik Zetterberg, Maxime Montembeault, Paolo Vitali, Kaj Blennow, Serge Gauthier, D. Louis Collins, Jesse Klostranec, Tharick A. Pascoal, Pedro Rosa‐Neto

**Affiliations:** ^1^ Translational Neuroimaging Laboratory, McConnell Brain Imaging Centre Montreal Neurological Institute Montreal Neurological Institute McGill University Montreal Quebec Canada; ^2^ Department of Neurology and Neurosurgery McGill University Montreal Quebec Canada; ^3^ The McGill University Research Center for Studies in Aging Douglas Mental Health Institute Montreal Quebec Canada; ^4^ Translational Neurodegeneration Laboratory, Department of Neurology University Medical Center Hamburg‐Eppendorf Hamburg Germany; ^5^ Institute of Neuroimmunology and Multiple Sclerosis, University Medical Center Hamburg‐ Eppendorf Hamburg Germany; ^6^ Lawrence Berkeley National Laboratory Berkeley California USA; ^7^ Department Psychiatry, School of Medicine University of Pittsburgh Pittsburgh Pennsylvania USA; ^8^ Department of Radiology Perelman School of Medicine at the University of Pennsylvania Philadelphia Pennsylvania USA; ^9^ Department of Psychiatry and Neurochemistry Institute of Neuroscience and Physiology, The Sahlgrenska Academy, University of Gothenburg Mölndal Sweden; ^10^ Department of Neurology, School of Medicine University of Pittsburgh Pittsburgh Pennsylvania USA; ^11^ Wallenberg Centre for Molecular Medicine University of Gothenburg Gothenburg Sweden; ^12^ King's College London Institute of Psychiatry, Psychology and Neuroscience, Maurice Wohl Institute Clinical Neuroscience Institute London UK; ^13^ NIHR Biomedical Research Centre for Mental Health and Biomedical Research Unit for Dementia at South London and Maudsley NHS Foundation London UK; ^14^ Clinical Neurochemistry Laboratory Sahlgrenska University Hospital Mölndal Sweden; ^15^ Department of Neurodegenerative Disease UCL Institute of Neurology, Queen Square London UK; ^16^ UK Dementia Research Institute at UCL London UK; ^17^ Department of Biomedical Engineering McGill University Montreal Quebec Canada; ^18^ Paris Brain Institute, ICM, Pitié‐Salpêtrière Hospital, Sorbonne University Paris France; ^19^ Neurodegenerative Disorder Research Center, Division of Life Sciences and Medicine, and Department of Neurology Institute on Aging and Brain Disorders University of Science and Technology of China and First Affiliated Hospital of USTC Hefei P.R. China; ^20^ Hong Kong Center for Neurodegenerative Diseases Clear Water Bay Hong Kong China; ^21^ Wisconsin Alzheimer's Disease Research Center University of Wisconsin School of Medicine and Public Health, University of Wisconsin–Madison Madison Wisconsin USA; ^22^ Centre for Brain Research Indian Institute of Science Bangalore India; ^23^ Department of Diagnostic and Interventional Neuroradiology Montreal Neurological Institute and Hospital McGill University Montreal Quebec Canada; ^24^ Departments of Neurology and Radiology The Peter O'Donnell Jr. Brain Institute (OBI) University of Texas Southwestern Medical Centre (UTSW) Dallas Texas USA

**Keywords:** Alzheimer disease, amyloid‐beta, biomarkers, brain ventricles, cerebrospinal fluid, choroid plexus, cognition disorders, glial fibrillary acidic protein, neurodegenerative diseases, positron‐emission tomography, tau proteins

## Abstract

**INTRODUCTION:**

Enlargement of the choroidal–ventricular system occurs in aging and Alzheimer's disease (AD), but emerging evidence links these abnormalities to amyloid beta (Aβ) aggregation. We tested this hypothesis by assessing associations between AD pathophysiology and choroidal–ventricular system measures across the AD continuum.

**METHODS:**

Ventricular volume, choroid‐plexus volume, and ventricular radioactivity after positron emission tomography (PET) tracer injections were analyzed in 385 Translational Biomarkers in Aging and Dementia (TRIAD) and 282 Alzheimer's Disease Neuroimaging Initiative (ADNI) participants using linear models and partial correlations. A composite score combining these measures was also tested against established AD biomarkers.

**RESULTS:**

With advancing AD stages, ventricular and choroid‐plexus volumes increased while ventricular radioactivity declined. These measures were interrelated, and abnormalities appeared even in amyloid‐negative elderly. Across cohorts, they correlated with amyloid‐ and tau‐PET, cerebrospinal fluid (CSF) and plasma p‐tau isoforms, glial fibrillary acidic protein (GFAP), and cognition. Voxel‐wise analyses showed strong associations with cortical Aβ, mediating downstream tau effects.

**DISCUSSION:**

Changes in the choroidal–ventricular system are mutually correlated and carry an additive‐effect on cortical Aβ load.

## Highlights

- The choroidalventricular composite score integrates PET and MRI measures.
- Abnormalities in this composite score emerge in aging before clinical Alzheimer's disease onset.
- Strong associations with Aβ PET, and biomarkers related to Aβ deposition, namely CSF and plasma p‐tau isoforms and GFAPs.
- Links with amyloid burden exceed those with hippocampal atrophy.
- The composite score may guide risk stratification and CSF clearance–targeted trials.

## 1 INTRODUCTION

The choroidal‐ventricular system encompasses a series of structures associated with cerebrospinal fluid (CSF) dynamics, including production and flow. This system plays a critical role in maintaining brain homeostasis by cushioning neural tissue, facilitating metabolic waste clearance, and regulating the chemical environment ^1^. CSF circulates through the ventricular system into the subarachnoid space, where it is subsequently either incorporated into the brain extracellular fluid via perivascular spaces or reabsorbed into the venous system, supporting molecular exchange between the brain and periphery ^2^. Disruptions in the choroidal‐ventricular system can potentially impair waste removal and immune cell trafficking and have been implicated in neurodegenerative processes ^3^. In Alzheimer's disease (AD), the progressive accumulation of amyloid‐beta (Aβ) begins decades before clinical symptoms ^4^, ^5^. Emerging evidence suggests that impaired abnormalities in the choroidal‐ventricular system may contribute to Aβ retention, creating a permissive environment for downstream amyloid aggregation ^6^, ^7^, ^8^, ^9^. More recently, Tijms et al. ^10^ used CSF proteomics in AD to define five molecular subtypes with distinct biological pathways and genetic risk profiles. Among them, Subtype 4 (choroid plexus dysfunction) was characterized by protein changes linked to impaired choroid plexus function, including reduced transport, barrier integrity, and CSF production pathways. Genetic associations suggested involvement of variants affecting epithelial and barrier processes. Clinically, this subtype showed structural alterations in the choroid plexus and ventricular system, indicating that CSF clearance and barrier dysfunction may play a key role in disease progression.

Directly measuring choroidal–ventricular changes in vivo remains challenging, prompting reliance on structural and functional proxies. For example, the choroid plexus (there are, in fact, numerous locations where they can be found) is the primary site of CSF production, and its volume has drawn attention as a potential biomarker of choroidal‐ventricular dysfunction. Their morphological changes, including volumetric enlargement, have been associated with reduced CSF secretion, Aβ42 clearance decline, and cognitive impairment ^11^, ^12^, ^13^, ^14^. Ventricular CSF radioactivity measured with positron emission tomography (PET) has been recently proposed as a surrogate for choroid plexus function ^15^. As there are no binding sites for PET tracers in the cerebrospinal fluid, ventricular radioactivity has been interpreted as the result of transport of tracer from the plasma to the CSF via the choroid plexus ^16^. Finally, ventricular dilation, which is another component of the choroidal–ventricular system, has long been conceptualized as a result of brain neurodegeneration and has also been related to amyloid aggregation ^17^, ^18^. Yet, ventricular enlargement also occurs during normal aging and may reflect early compensatory changes in CSF circulation, preceding overt atrophy and clinical symptoms ^19^, ^20^. Although these features (choroid plexus volume, ventricular volume, and ventricular radioactivity) are individually associated with AD pathology, each one captures distinct aspects of the choroidal–ventricular clearance system. Indeed, the associations between these choroid–ventricular system abnormalities have never been assessed simultaneously in the same population.

In an aging population, a substantial variability in the prevalence of the brain choroidal–ventricular system has been reported. To better understand the potential contributions of the choroidal–ventricular clearance system to AD pathological changes, we assessed the associations between distinct segments of the choroidal‐ventricular system as a function of AD pathophysiology. As such, we also devised a composite metric that encompasses ventricular and choroid plexus changes with a PET‐based functional proxy of CSF flow. By combining these three markers together, we aim to derive a better proxy of abnormalities of the choroidal‐ventricular. We hypothesized that the composite score abnormalities might reflect early Aβ aggregation and correlate with clinical, cognitive, CSF, and plasma biomarkers across the AD continuum.

## 2 MATERIAL AND METHODS

### 2.1 Participants

In the current study, we utilized data from the Translational Biomarkers in Aging and Dementia (TRIAD) cohort at McGill University ^21^. Additionally, all analyses were replicated in the Alzheimer's Disease Neuroimaging Initiative (ADNI) cohort (https://adni.loni.usc.edu/), including individuals classified as cognitively unimpaired (CU) or cognitively impaired (CI). We included 472 individuals from the TRIAD cohort, comprising 52 young individuals under 25 years of age (CU[Y]) and 267 CU older adults, as well as 59 individuals with mild cognitive impairment (MCI) and 94 individuals with AD dementia, both groups with confirmed amyloid‐positive status. Exclusion criteria included individuals with non‐AD dementias, such as frontotemporal dementia or non‐AD dementia, along with participants not on the AD clinicopathologic continuum. Additionally, we systematically excluded participants with signs of hydrocephalus, defined by an Evans' Index greater than 0.3^22^. Among the 472 participants, 385 underwent [^18^F]MK6240‐PET imaging, 392 received [^18^F]AZD4694‐PET imaging, and 403 had magnetic resonance imaging (MRI). All individuals included in the group comparisons and voxel‐wise analyses had amyloid, tau‐PET scans, and MRI. Based on these inclusion criteria, the final sample consisted of 385 participants (Supplemental Table 1).

The ADNI cohort included 740 participants. Applying the same inclusion criteria as TRIAD and ensuring all participants had amyloid‐PET ([^18^F]AV45) and tau‐PET ([^18^F]AV1451) scans, and MRI studies. The final sample comprised 282 participants (Supplemental Table 2).

CU participants included young individuals under 25 years old (CU[Y]) (only in TRIAD) and older adults with a Clinical Dementia Rating (CDR) score of 0, no objective cognitive impairment, and preserved activities of daily living (ADL). CI participants included individuals with MCI or AD dementia, defined by CDR = 0.5 and relatively presented ADL (MCI) or CDR = 1–2 with ADL impairment (AD dementia). Additional cohort details, including inclusion and exclusion criteria, have been previously published for TRIAD ^21^ and ADNI [https://adni.loni.usc.edu/].

**RESEARCH IN CONTEXT**

1. **Systematic review**: A growing body of literature has proposed that alterations in the brain compartments associated with CSF facilitate Aβ aggregation, due to reduced Aβ clearance. However, such investigations generally tested individual parameters. PubMed and selective conference abstract searching identified few investigations that combined structural, functional, and molecular evaluation of the choroidal–ventricular system.
2. **Interpretation**: Using two independent large cohorts, we show that ventricular volume, choroid plexus volume, and ventricular radioactivity after a PET tracer injection are highly intercorrelated and independently associated with brain amyloid‐beta, but not tau load. We also showed that a composite score integrating these measures is strongly associated with amyloid‐PET, CSF and plasma biomarkers (including p‐tau isoforms and GFAP). Importantly, the composite score had stronger associations with amyloid‐beta burden than with hippocampal atrophy, distinguishing early clearance dysfunction from downstream neurodegeneration.
3. **Future directions**: Longitudinal validation, integration into risk models, and application in prevention and treatment trials are needed.

TRIAD participants were consecutively recruited from the community or the McGill Centre for Studies in Aging memory clinic between November 2016 and March 2024. ADNI participants in this study were enrolled from multiple sites across North America between December 2016 and February 2022. The TRIAD study was approved by the Montreal Neurological Institute (MNI) PET Working Committee and the Douglas Mental Health University Institute Research Ethics Board. The ADNI study received approval from the institutional review boards of all participating institutions. All participants provided written informed consent.

### 2.2 Imaging acquisition and processing

#### 2.2.1 TRIAD

A high‐resolution 3 Tesla Siemens MAGNETOM MRI scanner with a standard head coil acquired T1‐weighted images via an ultrafast gradient echo 3D sequence (isotropic 1 mm voxels, TR: 2,300 ms, TE: 2.96 ms, FoV: 256 mm, flip angle: 9°). [^18^F]AZD4694 and [^18^F]MK6240‐PET scans were obtained using a high‐resolution brain‐dedicated Siemens High Resolution Research Tomograph. Participants received a radioactive dose activity of 185–260 MBq per scan. Tau‐PET images were acquired 90–110 minutes post‐injection of [^18^F]MK6240, and Aβ‐PET images 40–70 minutes post‐injection of [^18^F]AZD4694. Image reconstruction was performed using a sequential subset expectation‐maximization algorithm. A 6‐minute transmission scan using a rotating ^137^Cs point source accounted for motion, dead time, decay, random coincidences, and scatter.

#### 2.2.2 ADNI

Comprehensive ADNI neuroimaging acquisition and pre‐processing protocols are available online [https://adni.loni.usc.edu/]. Tau‐PET images were acquired 75–105 minutes post‐injection of [^18^F]AV1451, and Aβ‐PET images 50–70 min post‐injection of [^18^F]AV45.

### 2.3 Derivation of standardized uptake value ratio

#### 2.3.1 TRIAD

PET images were registered to their T1‐weighted MRI native space using ANTs (version 2.2.0) before linear and nonlinear transformation into ADNI template space. PET images were smoothed to 8 mm full‐width half‐maximum (FWHM). A meningeal mask was applied to minimize spillover artifacts ^23^. Standardized uptake value ratio (SUVR) for [^18^F]MK6240‐PET was calculated using the inferior cerebellum gray matter as the reference region ^24^, while for [^18^F]AZD4694, the entire cerebellum gray matter was used ^25^. The global [^18^F]AZD4694 SUVR composite was derived from the neocortex, encompassing the precuneus, prefrontal, orbitofrontal, parietal, temporal, and cingulate cortices ^25^. The global [^18^F]MK6240 SUVR was obtained from a temporal meta‐region of interest (ROI), which included the entorhinal cortex, amygdala, hippocampus, and the parahippocampal, fusiform, lingual, inferior temporal, and middle temporal gyri ^26^. Aβ positivity was defined as global [^18^F]AZD4694 SUVR > 1.55^25^ and tau positivity was defined as temporal [^18^F]MK6240 SUVR > 1.18^20^.

#### 2.3.2 ADNI

Pre‐processed ADNI PET images followed the same processing steps and SUVR derivation protocol as TRIAD. In ADNI, Aβ positivity was defined as global [^18^F]AV45 SUVR > 1.11^27^, and tau positivity was defined as global [^18^F]AV1451 SUVR > 1.35^28^.

### 2.4 Biomarkers

A subset of participants underwent CSF and plasma assessments in both samples as previously described [^25,29^ http://adni.loni.usc.edu/data‐samples/clinical‐data/]. This includes CSF and Plasma Aβ40 and 42, phosphorylated tau (p‐tau)s [181, 217, and 231], glial fibrillary acidic protein (GFAP) and total Tau (t‐tau) in TRIAD. In the TRIAD cohort, plasma Aβ40, Aβ42, p‐tau181, and GFAP were quantified using the Simoa® platform (Quanterix), with plasma p‐tau217 measured by the validated ALZpath assay; CSF Aβ40, Aβ42, p‐tau181, and total tau were measured on the Lumipulse® G platform (Fujirebio), while CSF p‐tau217 and p‐tau231 were assessed using single molecule assay (Simoa) ‐based immunoassays.

PET‐based Braak staging was assigned using SUVRs extracted from predefined Braak ROIs^20^, ^23^. Thresholds were determined using 2.5 standard deviation (SD) over CU(Y) participants in TRIAD cohort. We used 10 CU(Y) from the TRIAD cohort with [^18^F]AV1451‐PET scan as the reference group for ADNI. The hippocampus was excluded from the Braak I and II ROI due to significant off‐target binding in the choroid plexus ^30^. Participants not following hierarchical Braak staging were excluded.

### 2.5 Volumetric assessments

Lateral ventricle volume, choroid plexus volume, hippocampal volume, total gray matter and white matter volume, along with the total intracranial volume (ICV) were extracted from native MRI space using FreeSurfer (version 6.0). Segmentation ensured accurate differentiation between gray matter, white matter, and CSF regions. ICV was used to normalize all extracted brain regions volume.

### 2.6 Derivation of ventricular radioactivity

Ventricular radioactivity was quantified from amyloid‐PET imaging using [^18^F]AZD4694 in TRIAD and [^18^F]AV45 in ADNI. After standard preprocessing (motion correction, registration to each subject's T1‐weighted MRI), lateral ventricles were defined as ROIs using subject‐specific FreeSurfer segmentation masks, with the choroid plexus region excluded to minimize non‐specific binding and partial‐volume contamination from adjacent tissue ^30^. The mean SUVR within the ventricular ROI was extracted to represent ventricular radioactivity for each participant. This measure reflects the level of tracer diffusing into the ventricular CSF and is considered a surrogate of CSF clearance efficiency and choroid plexus‐mediated transport.

### 2.7 Statistical analysis

One‐way analysis of variance (ANOVA) was conducted in Python using the SciPy library, followed by Tukey's post‐hoc test using the stats model's library, for group comparisons based on A/T stages. Scatter plots included regression lines, p‐values, and Spearman correlation coefficients, and were generated using an in‐house Python code. P‐values were adjusted for multiple comparisons across all regions using the false discovery rate (FDR) method (*q* < 0.05). Voxel‐based linear regression analysed the relationship between the choroidal‐ventricular parameter composite score and PET using MATLAB VoxelStats ^31^, adjusting for age, sex, apolipoprotein E4 (APOE4) status, as well as total gray matter volume. Multiple comparisons were corrected using the random field theory method ^32^ (*P* < 0.001).

All extracted brain's regions volumes, including choroid plexus, ventricular volumes, hippocampal volume, total gray matter and white matter volume, were adjusted for ICV using linear regression and residuals were used in all analysis to control for head size variability.

The composite score was calculated by averaging the *Z* scores of three parameters per subject using the following equation:

$$\frac{ventricular\;volume_{corrected}Z+choroid\;plexus\;volume_{corrected}Z+ventricular\;radioactivity_{reversed}Z}{3}$$

*Z* scores were calculated based on the mean and SD of Young participants normalized in TRIAD and CU A‐T‐ group's mean and SD in ADNI. Ventricular radioactivity values were inverted (by multiplying its *z*‐scored values by −1), as they decrease in the context of AD^15^, ^16^.

Smoothed trajectories of biomarker variation across Braak stages were modelled using locally estimated scatterplot smoothing (LOESS) regression. To enable comparison between biomarkers with different scales and units, all biomarker values were standardized (z‐scored across the entire dataset), yielding the “standardized biomarker abnormality” scale shown on the y‐axis. Inflection points in the trajectories were identified within the same LOESS model through visual inspection and verified by computing the first derivative of the fitted curve to locate local maxima and minima, corresponding to the early and later phases of AD.

## 3 RESULTS

The final sample included 667 participants, comprising 51 young individuals under 25 years old (CU[Y]), 166 A−T− older adults, 64 A+T−, 5 A−T+, and 99 A+T+ from the TRIAD cohort, as well as 185 A−T− older adults, 66 A+T−, 2 A−T+, and 29 A+T+ from the ADNI cohort. Across both cohorts, 180 participants returned for longitudinal follow‐up, contributing imaging and clinical data spanning up to 6 years. Supplemental Table 1 and 2 summarizes the full demographics of participants from TRIAD and ADNI cohorts included in the current study. We found no significant differences in years of education (*p* = 0.32) or age (*p* = 0.21). MCI and AD participants showed significantly lower Mini‐Mental State Examination (MMSE) (*p* < 0.01) and CSF Aβ42 (*p* < 0.001) and significantly higher phosphorylated tau (p‐tau) (*p* < 0.001).

### 3.1 The choroidal–ventricular parameters are associated with AD pathophysiological severity

In TRIAD, our cross‐sectional analysis revealed significant group effects for ventricular volume (*F*(3, 380) = 76.99, *η^2^* = 0.40, *p* < 0.001) and choroid plexus volume (*F*(3, 380) = 65.99, *η^2^* = 0.37, *p* < 0.001). Compared with CU(Y), A‐T‐ showed larger ventricular volume (+2.17 Z, Cohen's *d* = 1.85, *p* < 0.001) and larger choroid plexus volume (+2.06 Z, *d* = 1.65, *p* < 0.001). A+T‐ exhibited a further increase in ventricular volume relative to A−T− (+0.75 Z, *d* = 0.54, *p* = 0.010) and an increase in choroid plexus volume (+0.89 Z, *d* = 0.43, *p* = 0.04). A+T+ showed an additional increase in ventricular volume over A+T− (+1.25 Z, *d* = 0.66, p < 0.001) and a significant increase in choroid plexus volume (+0.74 Z, *d* = 0.61, *p* = 0.002). For ventricular radioactivity (F(3, 380) = 27.90, *η^2^* = 0.20, *p* < 0.001), A‐T‐ had higher values than CU(Y) (+1.51 Z, *d* = 1.23, *p* < 0.001), A+T− was similar to A−T− (*p* = 0.980), and A+T+ had lower values than A+T− (−0.69 Z, *d* = 0.47, *p* = 0.011).

**FIGURE 1:**
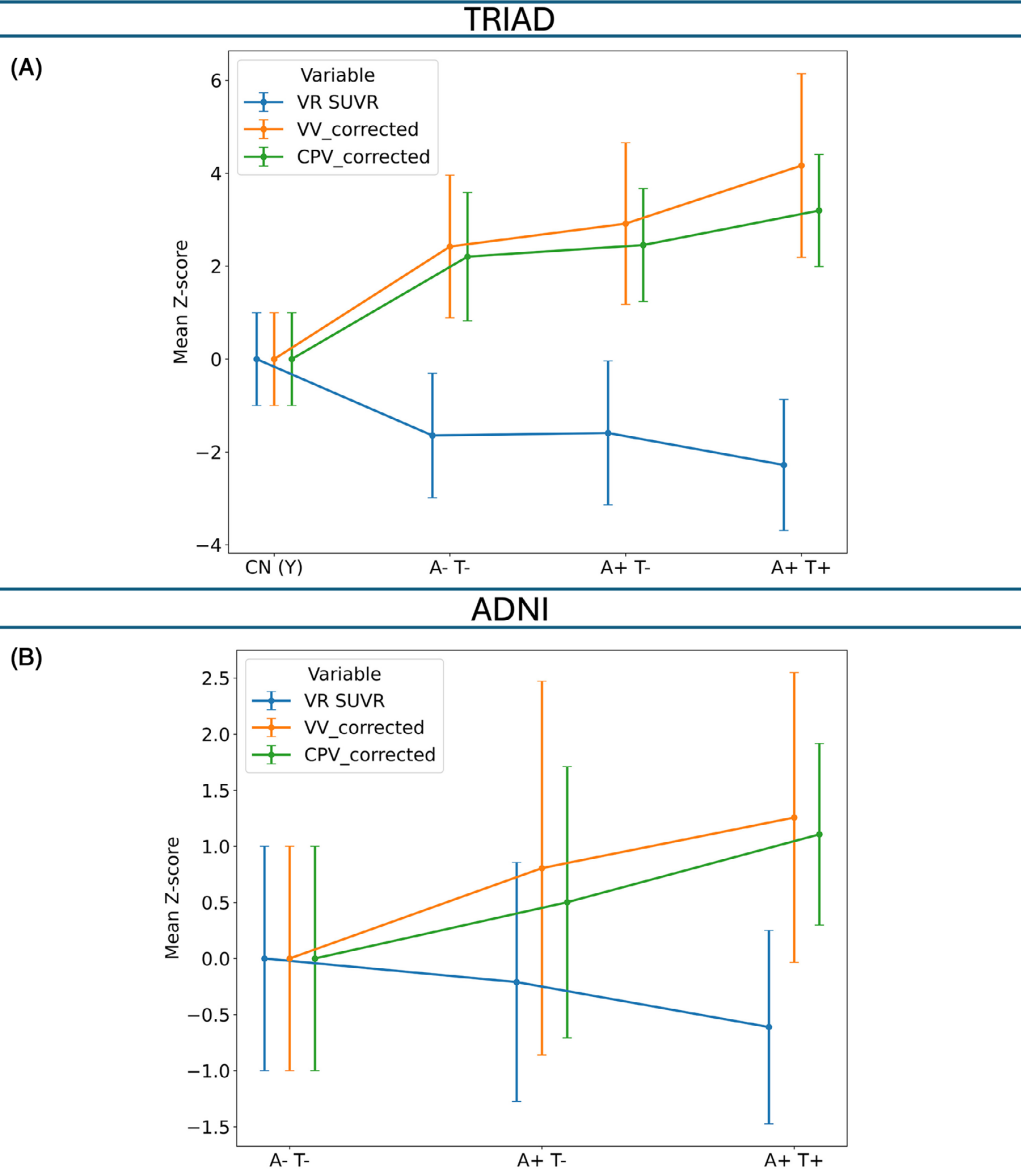
The overall trend of cerebrospinal fluid (CSF) clearance system parameter abnormality across A/T stages. CSF clearance system parameters including choroid plexus volume (CPV_corrected) and ventricular volume (VV_corrected), both corrected for intracranial volume (ICV), and ventricular radioactivity (VR) standardized uptake value ratio (SUVR) were *Z* scored and normalized based on the mean and standard deviation (SD) of Young Translational Biomarkers in Aging and Dementia (TRIAD) or cognitively unimpaired (CU) A−T− Alzheimer's Disease Neuroimaging Initiative (ADNI). CPV_corrected and VV_corrected enlargement and VR SUVR decline started due to aging and further abnormality observed due to Alzheimer's disease (AD) in the presence of amyloid‐beta (Aβ) and tau, in both cohorts.

In ADNI, we also found that significant group effects for ventricular volume (F(2, 282) = 19.70, *η^2^* = 0.13, *p* < 0.001) and choroid plexus volume (*F*(2, 282) = 16.92, *η^2^* = 0.11, *p* < 0.001). Relative to A−T−, A+T− showed larger ventricular volume (0.81 Z, Cohen's *d* = 0.68, *p* < 0.001) and larger choroid plexus volume (0.50 Z, *d* = 0.48, *p* < 0.001); A+T+ vs A+T− showed a larger ventricular volume (0.65 Z, *d* = 0.59, *p* = 0.01) and larger choroid plexus volume (0.61 Z, *d* = 0.56, *p* = 0.02), and compared with A−T−, A+T+ had markedly larger ventricular volume (1.26 Z, *d* = 1.20, *p* < 0.001) and choroid plexus volume (1.11 Z, *d* = 1.13, *p* < 0.001). For ventricular radioactivity (*F*(2, 282) = 5.00, *η^2^* = 0.04, *p* = 0.007), A+T+ was lower than A−T− (−0.61 Z, *d* = 0.62, *p* = 0.007). These choroidal–ventricular group comparisons were observed in both cohorts (Figure 1).

### 3.2 Choroidal and ventricular volumes, as well as ventricular radioactivity, are associated with protein aggregation

Our Spearman correlation analysis revealed that all parameters were associated with global Aβ and temporal tau. Ventricular volume and choroid plexus volume correlated with global Aβ SUVR (TRIAD: *ρ* = 0.51 and 0.43; ADNI: *ρ* = 0.31 and 0.24; all *p* < 0.001). Ventricular radioactivity exhibited a negative correlation in TRIAD (*ρ* = −0.21; *p* < 0.001) but not in ADNI (*ρ* = 0.07; *p* = 0.20). Correlations with temporal tau were generally weaker than with Aβ but remained significant for most parameters in both cohorts (Supplementary Figure 1).

### 3.3 Choroidal and ventricular volumes, as well as ventricular radioactivity, are correlated

**FIGURE 2:**
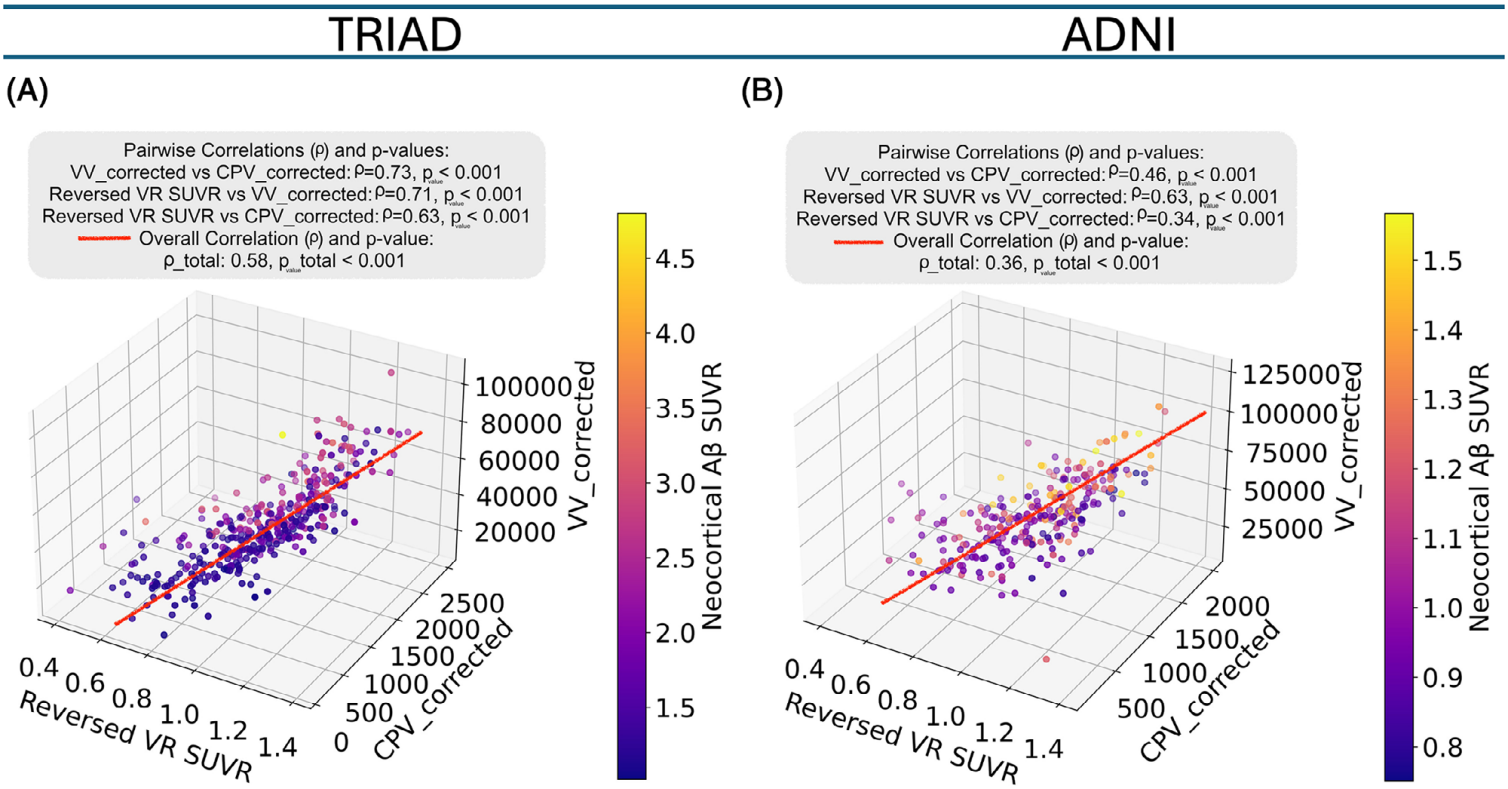
The choroidal–ventricular parameters exhibited strong inter‐correlations with one another. Choroid plexus volume (CPV_corrected), ventricular volume (VV_corrected), and ventricular radioactivity (VR) standardized uptake value ratio (SUVR) were highly correlated with each other. Additionally, they showed strong inter‐correlations, with an overall correlation of *β* = 0.58 (*p* < 0.001) in Translational Biomarkers in Aging and Dementia (TRIAD) and *β* = 0.36 (*p* < 0.001) in Alzheimer's Disease Neuroimaging Initiative (ADNI). In this figure, due to the overall decline in VR, we reversed it for better visualization.

The three CSF‐related ventricular parameters were interrelated, with overall correlations of 0.58 in TRIAD and 0.36 in ADNI (both *p* < 0.001). Pairwise comparisons further confirmed intercorrelations among all measures. These relationships are visualized in three‐dimensional plots (Figure 2).

### 3.4 The choroidal–ventricular parameter composite score is strongly associated with AD pathophysiology

**FIGURE 3:**
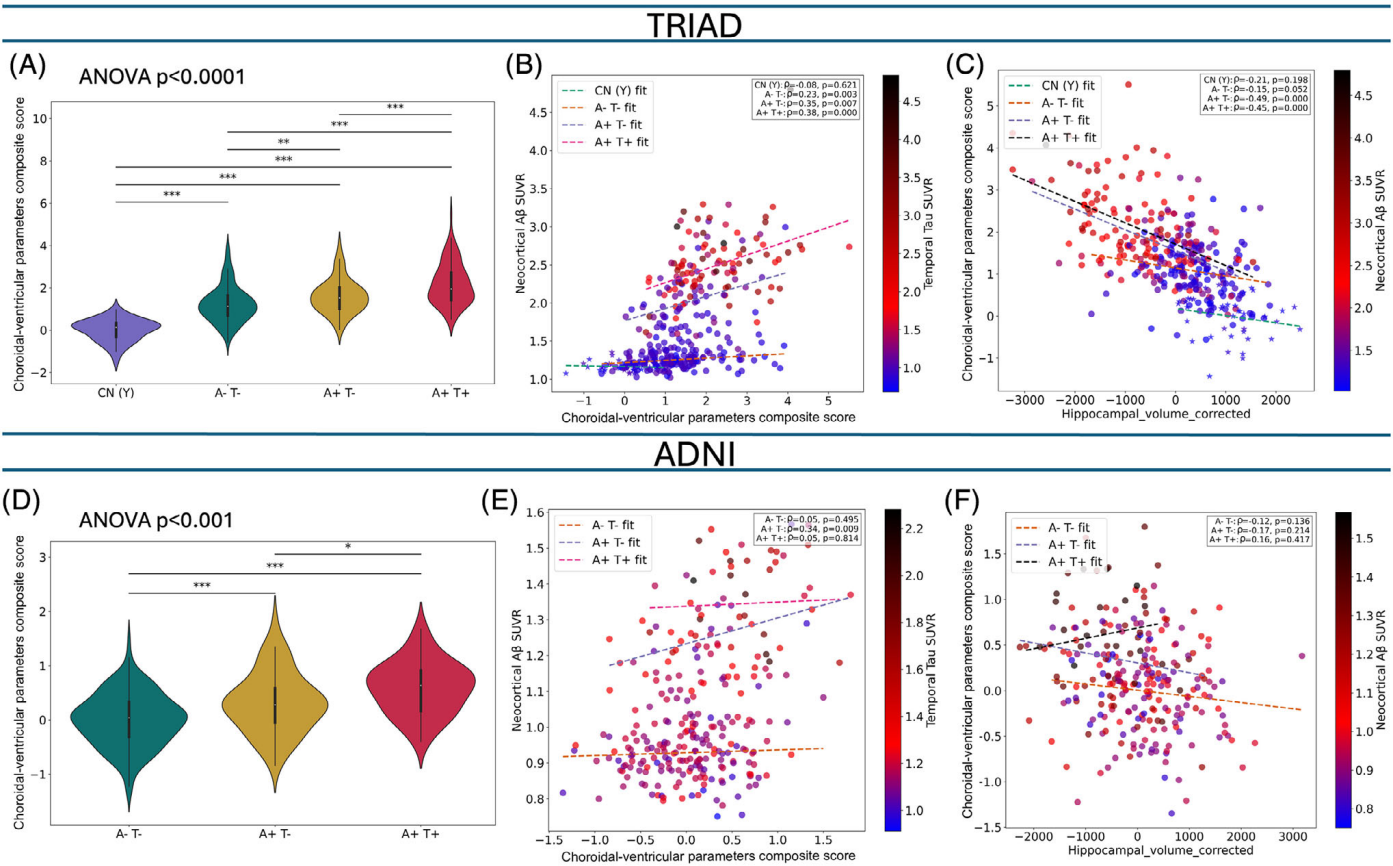
The choroidal–ventricular parameter composite score is strongly related to Alzheimer's disease (AD) pathophysiology. The choroidal–ventricular parameter composite score showed abnormality with aging and further alterations in AD across both cohorts with one‐way analysis of variance (ANOVA). "*", "**", and "***" represent Tukey post‐hoc tests following ANOVA for pairwise comparisons p‐values in the following ranges: 0.05 to 0.01, 0.01 to 0.001, and less than 0.001, respectively (A&D). Additionally, scatter plots show the relationship between the composite score and neocortical amyloid‐beta (Aβ) standardized uptake value ratio (SUVR) in the Translational Biomarkers in Aging and Dementia (TRIAD) cohort (B) and Alzheimer's Disease Neuroimaging Initiative (ADNI) cohort (E), stratified by biomarker‐defined groups: CU(Y) = cognitively normal young controls (green), A−T− (brown), A+T− (blue), and A+T+ (pink). Regression lines and Spearman correlation coefficients (r) with corresponding p‐values are displayed for each group. Spearman correlation *p*‐values were adjusted for multiple comparisons using the Benjamini–Hochberg false discovery rate (FDR) method, with *q* < 0.05 considered statistically significant. Point color intensity represents temporal tau SUVR, indicating higher tau burden in individuals with elevated choroidal–ventricular parameter composite scores and Aβ load. The composite score demonstrated strong positive associations with neocortical Aβ in preclinical and symptomatic A−T− groups, while no significant association was observed in CU(Y) individuals. Distinct patterns of association between the composite score and hippocampal volume (intracracial volume [ICV] ‐corrected) were observed across A/T stages (C&F), with no correlation in the A–T− group in TRIAD and no correlations across A/T stages in ADNI.

We observed marked abnormalities in the choroidal–ventricular parameter composite score across A/T stages in both cohorts. The composite score was correlated with global Aβ SUVR (TRIAD: *ρ* = 0.55; ADNI: *ρ* = 0.38), showing stronger associations than any individual parameter (Figure 3). Notably, the composite score remained associated (*ρ* = 0.23, *p* = 0.003) with Aβ load across all A/T groups, particularly in the A−T− group in TRIAD (representing normal elderly group) and in the A+T− group in ADNI (*ρ* = 0.43, *p* = 0.009) after multiple‐comparison correction.

To distinguish ventricular dilation driven by neurodegeneration from that associated with remodeling not linked to neuronal loss, we examined the associations between the composite score and hippocampal volume (ICV‐corrected) as a marker of neurodegeneration and observed a distinct pattern of association across A/T stages. We found no correlation in the A‐T‐ group in TRIAD and no correlation across A/T stages in ADNI (Figure 3C&F). Although hippocampal atrophy was evident across A/T stages in both cohorts, its association with Aβ SUVR was restricted to the A+T+ group in TRIAD (*ρ* = −0.36, *p* < 0.001) and was not significant in ADNI after multiple‐comparison correction (Supplementary Figure 2).

**FIGURE 4:**
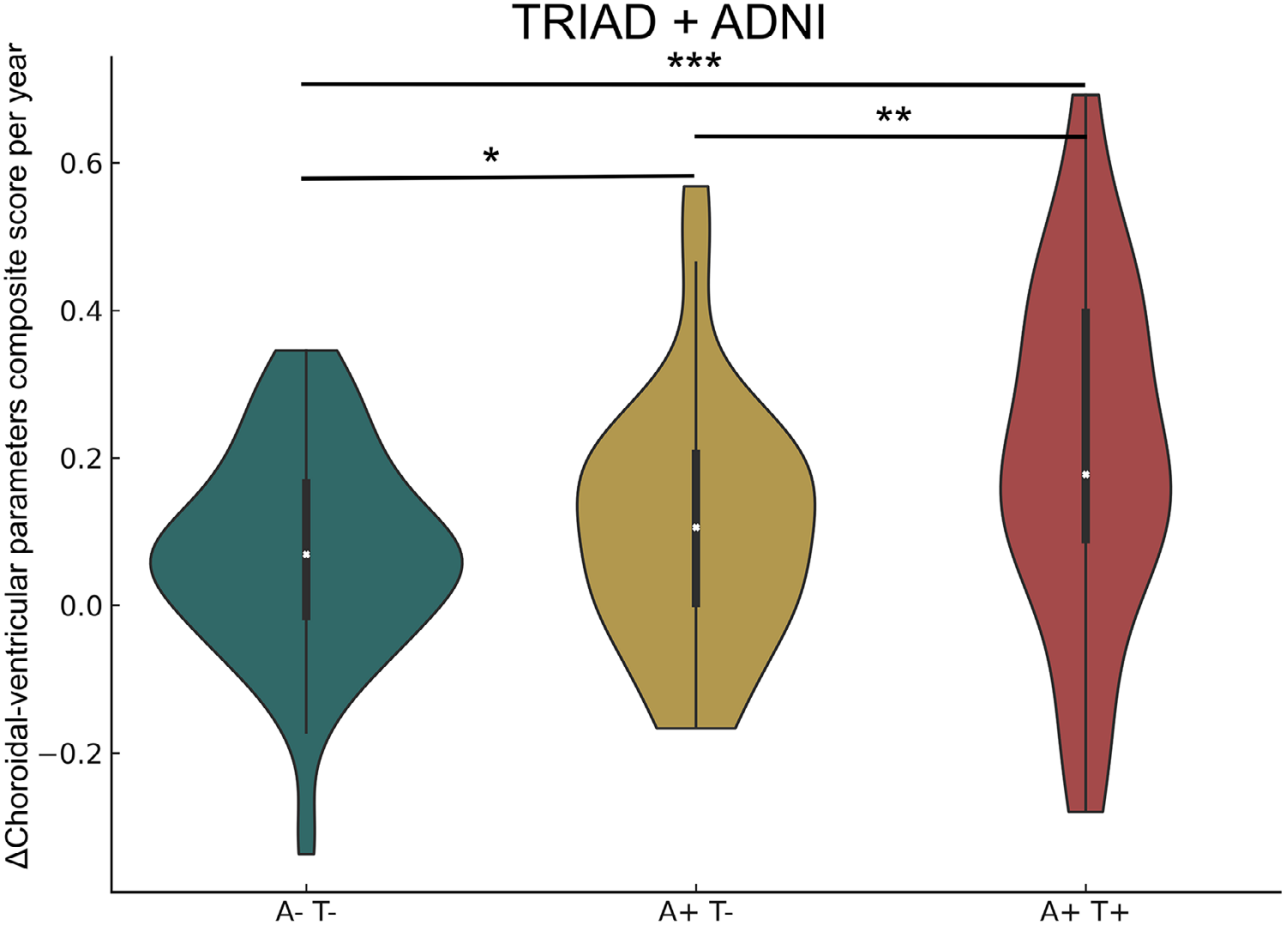
The choroidal–ventricular alterations evolve dynamically along the Alzheimer's disease (AD) continuum. The annualized change in the choroidal‐ventricular composite score (ΔComposite/year) across A/T stages in the combined Translational Biomarkers in Aging and Dementia plus Alzheimer's Disease Neuroimaging Initiative (TRIAD + ADNI) dataset increased stepwise from A−T− to A+T– and from A+T− to A+T+, indicating progressive choroidal‐ventricular remodeling along the AD continuum. "*", "**", and "***" represent Tukey post‐hoc tests following analysis of variance (ANOVA) for pairwise comparisons *p*‐values in the following ranges: 0.05 to 0.01, 0.01 to 0.001, and less than 0.001, respectively.

To further examine the dynamic evolution of these alterations, we calculated the annualized change in the choroidal‐ventricular composite score (ΔComposite/year) across A/T stages in the combined TRIAD + ADNI dataset. Among 667 participants, 180 returned for longitudinal follow‐up with imaging data spanning up to 6 years. This analysis revealed a significant stepwise increase in ΔComposite/year from A−T− to A+T− (*p* < 0.01) and from A+T− to A+T+ (*p* < 0.001) (Figure 4).

### 3.5 The choroidal–ventricular parameter composite score abnormalities are present in amyloid‐negative individuals

**FIGURE 5:**
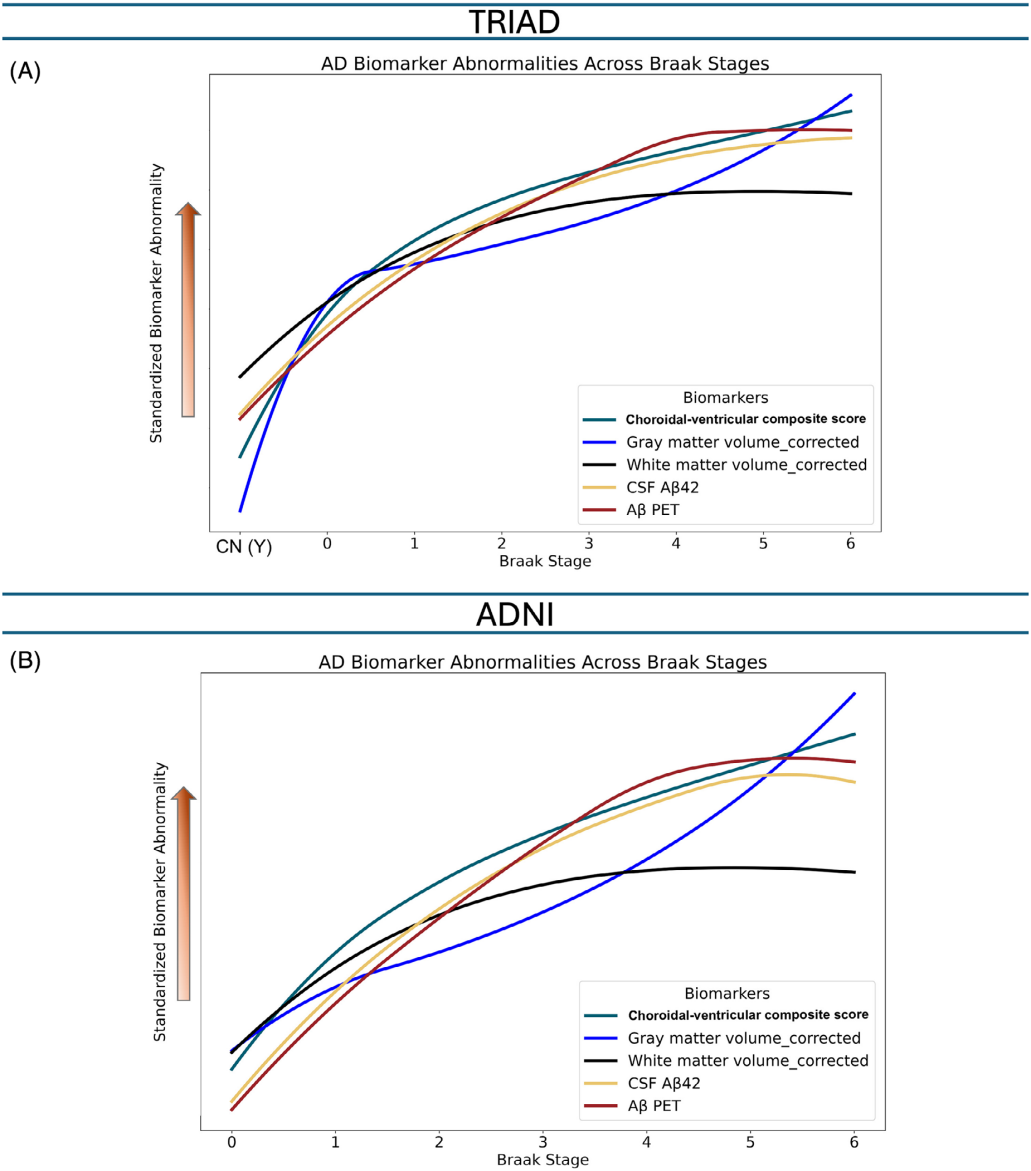
The choroidal–ventricular parameter composite score is an early change contributing to protein aggregation. In this conceptual framework, the x‐axis represents Braak stage rather than time. Unlike other Alzheimer's disease (AD) biomarker models, it does not depict a linear temporal progression of the disease. Instead, it illustrates various pathophysiological changes in relation to the spatial distribution of tau pathology as measured by tau‐positron emission tomography (PET). The y‐axis represents biomarker values standardized (*z*‐scored) across all participants to enable comparison between biomarkers. Inflection points were identified within the same locally estimated scatterplot smoothing (LOESS) fit and confirmed by local extrema of the first derivative. It is evident from this figure that the choroidal‐ventricular parameter composite score abnormality preceded cerebrospinal fluid (CSF) amyloid‐beta (Aβ) 42 decline as a marker of protein aggregation, and they are followed by Aβ‐PET standardized uptake value ratio (SUVR) abnormality.

LOESS analysis revealed that abnormalities in the choroidal–ventricular parameter composite score emerged earlier than declines in CSF Aβ42 and increases in Aβ‐PET SUVR. Two distinct inflection points were observed. The first occurred during normal aging (Braak 0), simultaneously occurring in the gray and white matter (before neurodegeneration caused by tauopathy, up to Braak 3), and coincided with the initial accumulation of Aβ. The second occurred in association with tau pathology (from Braak 3 and over), marking the onset of tau‐related neurodegeneration. At this stage, total white matter volume changes plateaued (similar to Aβ), while gray matter atrophy became more pronounced. Similar trajectories were observed across Braak stages in both cohorts (Figure 5).

### 3.6 The choroidal–ventricular parameter composite score associates with brain amyloid aggregates

**FIGURE 6:**
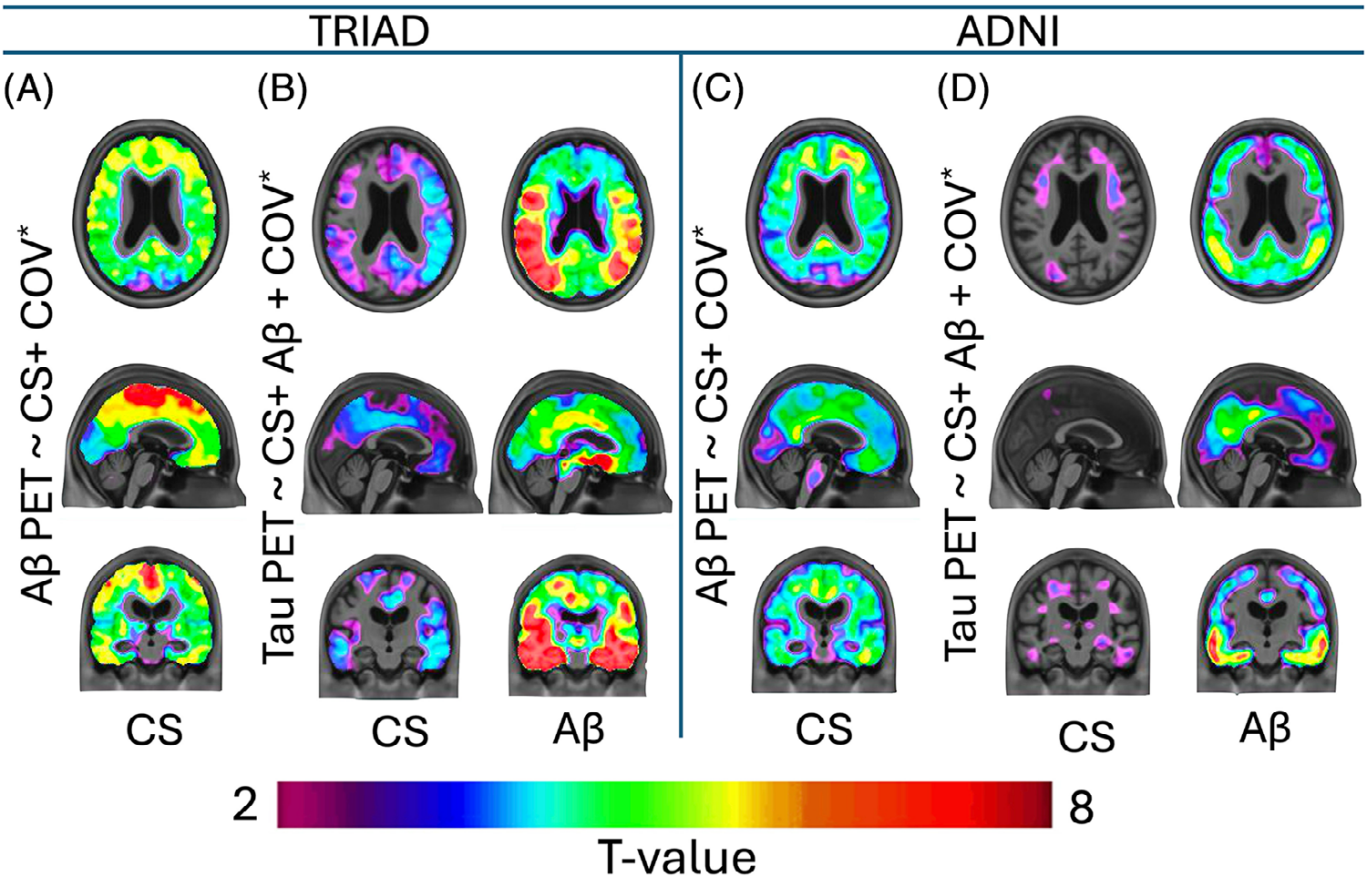
The choroidal–ventricular parameter composite score is associated with protein aggregation; however, amyloid‐beta (Aβ) carries the effect of it on tau. Voxel‐wise linear analysis for the older adult groups (65 years old and over, *N* = 265 in Translational Biomarkers in Aging and Dementia (TRIAD) and *N* = 244), false discovery rate (FDR)‐corrected for multiple comparisons at *P* < 0.001, visualized as T‐statistical parametric maps, and overlaid on a structural template indicated significant association of the composite score and Aβ‐positron emission tomography (PET) over the cortex. Additionally, its association with tau‐PET was significantly mediated by Aβ over the cortex. * Covariates: age, sex, apolipoprotein E4 (APOE4), gray matter volume.

Voxel‐wise linear modelling revealed that the choroidal–ventricular parameter composite score was significantly associated with Aβ load across widespread cortical regions. In this analysis we included gray matter volume as an additional key marker of neurodegeneration in AD to distinguish ventricular dilation driven by neurodegeneration from that arising through other mechanisms. In contrast, the association between the choroidal‐ventricular parameter composite score and tau load was not direct; instead, the effect was largely carried by Aβ, indicating a mediation pathway where amyloid burden drives the link between ventricular alterations and tau deposition. These patterns were highly consistent across both cohorts (Figure 6).

### 3.7 The choroidal–ventricular parameter composite score is associated with AD markers

**FIGURE 7:**
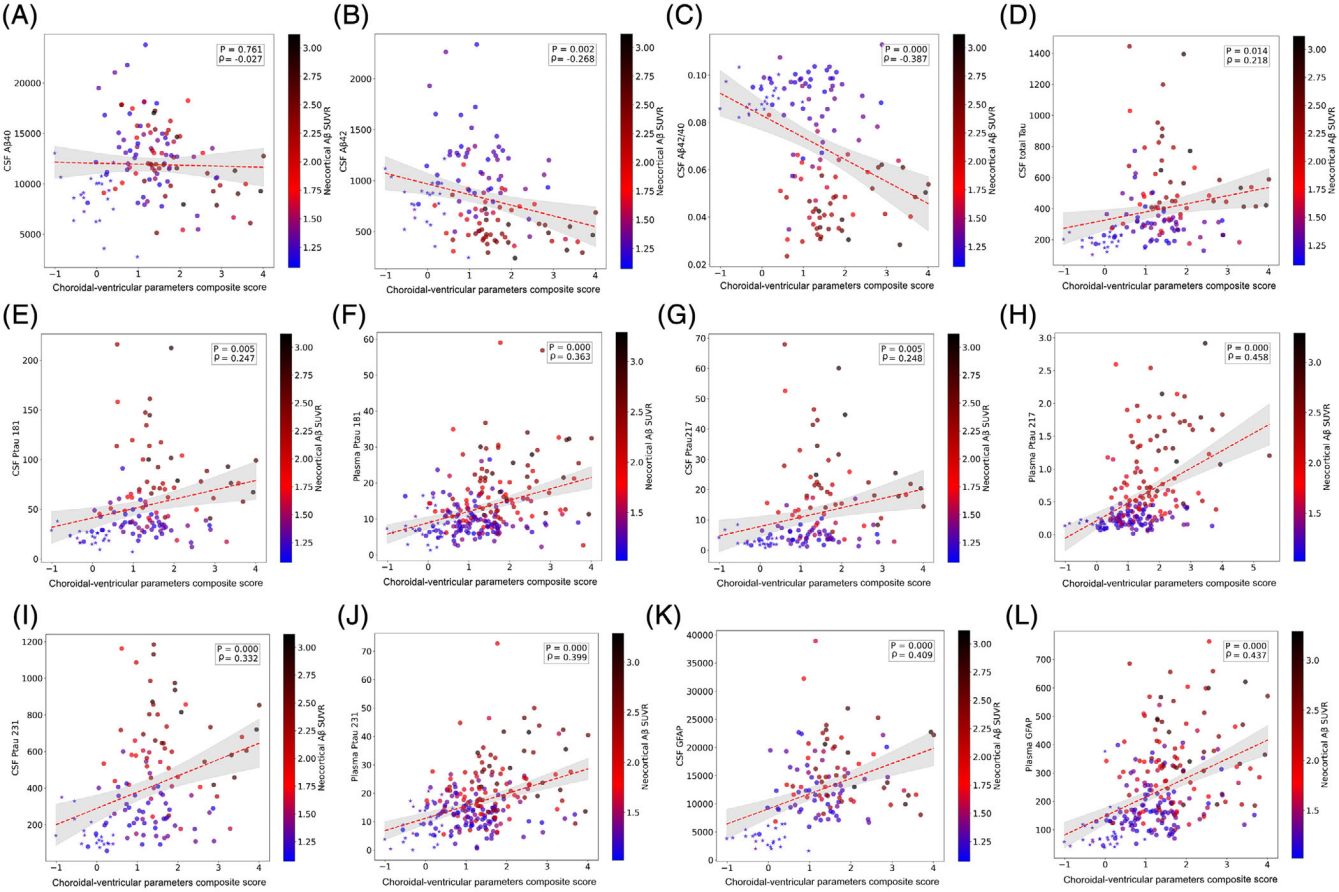
Almost all cerebrospinal fluid (CSF) and plasma biomarkers in Alzheimer's disease (AD) are correlated with the choroidal‐ventricular parameter score and showed the same trends (Translational Biomarkers in Aging and Dementia [TRIAD]). Scatterplots show correlations between the choroidal–ventricular composite score and:(A) CSF amyloid‐beta (Aβ) 40, (B) CSF Aβ42, (C) CSF Aβ42/40 ratio, (D) CSF total tau, (E) CSF p‐tau181, (F) plasma p‐tau181, (G) CSF p‐tau217, (H) plasma p‐tau217, (I) CSF p‐tau231, (J) plasma p‐tau231, (K) CSF glial fibrillary acidic protein (GFAP), and (L) plasma GFAP. Point colour indicates neocortical Aβ standardized uptake value ratio (SUVR; blue = lower, red = higher). Shaded areas represent 95% confidence intervals. CSF and plasma p‐taus (181, 217, 231) as sensitive and early marker of protein aggregation, GFAP as marker of astrocyte activation, and Aβ42 as marker of Aβ aggregation were all significantly correlated with the choroidal–ventricular parameter composite score.

**FIGURE 8:**
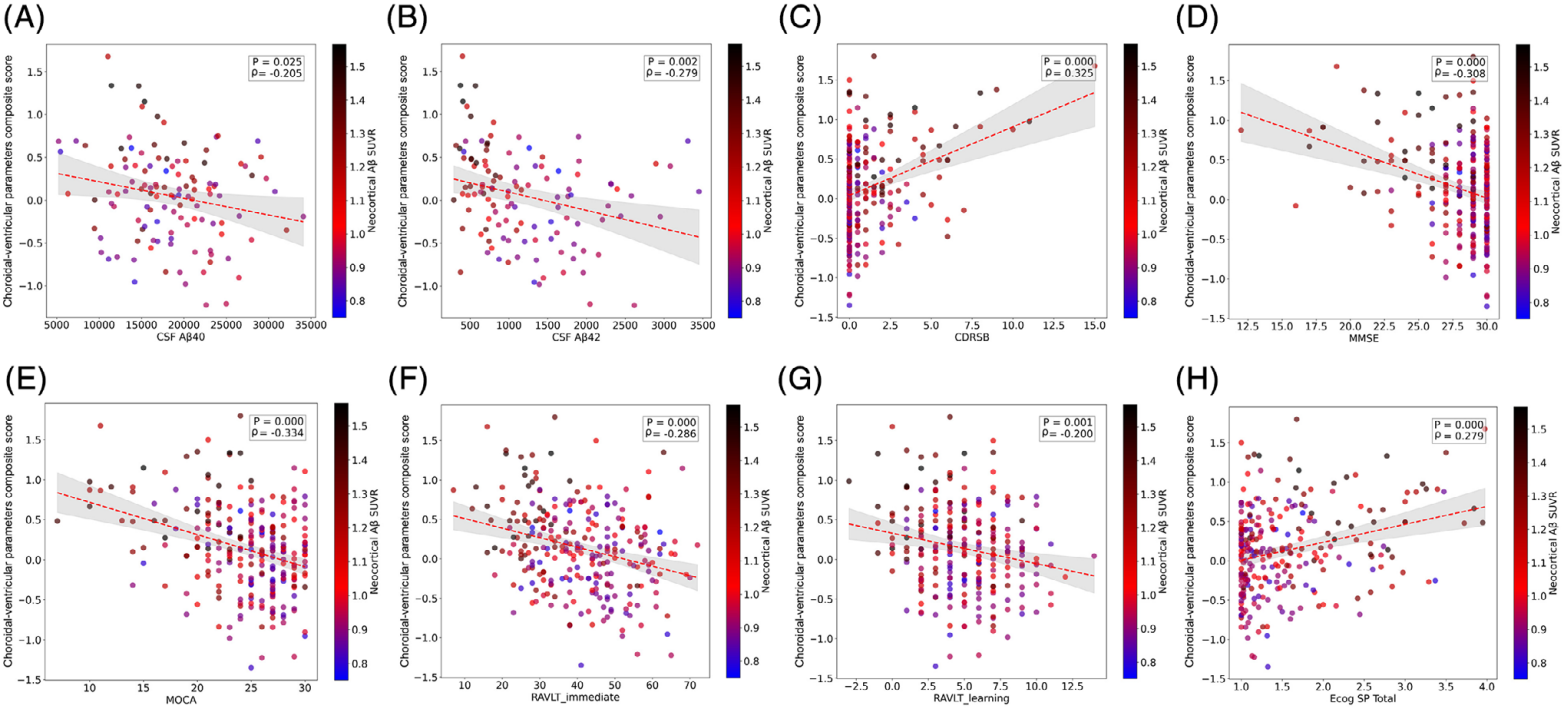
Almost all memory and cognitive scores are correlated with the choroidal‐ventricular parameter composite score and showed the same trends (Alzheimer's Disease Neuroimaging Initiative [ADNI]). Associations between the choroidal–ventricular parameter composite score and (A) cerebrospinal fluid (CSF) amyloid‐beta (Aβ) 40, (B) CSF Aβ42, (C) Clinical Dementia Rating–Sum of Boxes (CDR‐SOB), (D) Mini‐Mental State Examination (MMSE), (E) Montreal Cognitive Assessment (MoCA), (F) Rey Auditory Verbal Learning Test (RAVLT) immediate recall, (G) RAVLT learning, and (H) Everyday Cognition–Study Partner (Ecog‐SP) total score, with point colour indicating neocortical Aβ standardized uptake value ratio (SUVR; blue = lower, red = higher). Different memory and cognitive scores, including MMSE that assesses global cognitive function, MoCA as an early cognitive impairment marker, and CDR‐SOB which measures dementia severity by evaluating functional and cognitive decline across multiple domains, were significantly correlated with the composite score.

We finally examined correlations between the choroidal–ventricular parameter composite score and a wide range of AD‐related biomarkers, as well as memory and cognitive measures. As shown in Figure 7 (TRIAD) and Figure 8 (ADNI), the composite score was significantly associated with nearly all AD biomarkers, including CSF Aβ42 (but not Aβ40), the CSF Aβ42/40 ratio, multiple CSF and plasma p‐tau isoforms (181, 217, 231), CSF and plasma GFAP, and CSF t‐tau. Strong associations were also observed with global cognitive and memory performance, including the MMSE, Montreal Cognitive Assessment (MoCA), CDR‐Sum of Boxes (CDR‐SOB), and their respective sub‐domains.

## 4 DISCUSSION

We found that larger choroid plexus volume, greater ventricular volume, and reduced ventricular radioactivity after PET tracer injection were associated with early AD pathophysiology, particularly brain amyloidosis. These choroidal–ventricular metrics are mutually correlated and carry an additive effect on predicting cortical Aβ load. Our choroidal–ventricular score, combining these parameters, provided a better correlation with Aβ load as compared to its individual parts. Overall, these findings support our hypothesis that choroidal–ventricular function is also associated with brain amyloidosis in AD.

Our choroidal–ventricular parameter composite score is possibly influenced by multiple pathways, including CSF choroid plexus secretion, CSF ventricular circulation, perivascular exchange, and CSF absorption via both glymphatic and venous routes ^33^. It has been proposed that the disruption of this system compromises metabolic waste removal and is increasingly recognized as a pathogenic process in neurodegenerative diseases, particularly AD ^8^, ^34^. One of AD's defining features is the gradual extracellular accumulation of Aβ, which can precede clinical symptoms by decades and may create an environment that fosters tau aggregation and subsequent neurodegeneration ^35^, ^36^. Unravelling the early pathophysiological changes that facilitate this cascade is therefore crucial for devising novel therapeutic strategies.

In the present study, we introduce a novel composite metric integrating the choroid plexus volume, ventricular volume, and ventricular radioactivity as a PET‐derived proxy of CSF dynamics and choroid plexus function. The multi‐dimensional metric revealed stronger associations with global Aβ‐ and tau‐PET SUVR than any individual parameter, suggesting that it captures a broader spectrum of clearance impairment. The emergence of abnormalities in the composite score in amyloid‐negative individuals indicates an early relationship with AD pathophysiology, voxel‐wise analysis revealed a strong cortical association with Aβ. In contrast, its link to tau was largely mediated by Aβ, indicating a hierarchical effect where impaired clearance first facilitates amyloid accumulation, which then promotes tau pathology. By contrast, hippocampal atrophy, a signature neurodegeneration, did not predict early Aβ changes. Correlations with plasma and CSF biomarkers (e.g., p‐tau isoforms, GFAP) and cognition highlight the systemic impact of impaired CSF clearance across all disease stages.

Choroid plexus is the primary site of CSF production and a key regulator of brain–periphery exchanges. Structural changes in choroid plexus have been noted across the AD continuum and could be associated with reduced CSF production and secretion, altered transporter function, and impaired barrier integrity ^11^, ^33^. These alterations may compromise the removal of soluble Aβ from the interstitial fluid, thereby fostering its extracellular accumulation ^8^, ^33^, ^34^. A growing body of evidence suggests that choroid plexus enlargement is associated with higher cortical Aβ load, lower CSF Aβ42 as well as poorer memory and executive function performance ^14^, ^37^, ^38^, ^39^. Inflammatory remodeling of the choroid plexus epithelium and disruption of perivascular exchange could create a favorable environment for early protein accumulation ^10^, ^40^, placing choroid plexus morphometry as both a biomarker and a potential mediator of impaired clearance in AD.

PET‐derived ventricular radioactivity has recently been introduced as a proxy of choroid plexus function through its link with CSF production. As the ventricular CSF lacks specific binding sites for Aβ or tau ligands, signal intensity following tracer injection reflects tracer transport from plasma into CSF mainly through the choroid plexus, rather than any type of specific, non‐specific, or off‐target bindings ^15^, ^16^. It has been proposed that reduced ventricular radioactivity reflects compromised choroid plexus function, impaired CSF clearance, increased Aβ brain load, and poorer memory and executive function performance in AD as compared to a same aged healthy adults’ group ^15^, ^16^. These associations corroborate a framework in which diminished ventricular radioactivity signals impaired CSF production and ultimately decrease brain CSF parenchymal flux in the glymphatic system, facilitating extracellular Aβ accumulation ^41^. It is noteworthy that the decline in tracer concentrations in the ventricle occurs despite a significant increase in the cortex, highlighting the opposite direction of tracer concentrations in different brain compartments.

Ventricular enlargement has been associated with disease progression and higher Aβ accumulation in AD ^41^. Ventricular enlargement may contribute to altered CSF hydrodynamics by reducing pulsatile flow amplitude, changing ependymal surface contact with CSF, and prolonging the residence time of solutes in the brain parenchyma ^42^, ^43^. This alteration by causing perturbation in the bulk of CSF flow, contribute to impaired clearance of soluble Aβ and promote its aggregation into fibrillar plaques. Additionally, recent studies pointed toward a notion that periventricular white matter changes associated with ventricular expansion may cause astrocyte activation and further impaired Aβ clearance by disruption in the glymphatic system pathways ^1^, ^44^.

In this study, we aimed to expand on these findings by showing that these components, though individually informative, are interdependent and collectively provide a more powerful indicator of clearance dysfunction. This supports a conceptual model where structural and functional CSF changes occur upstream of overt neurodegeneration, influencing Aβ kinetics before cognitive impairment manifests.

We reported that these parameters are strongly intercorrelated, reflecting their shared role in CSF clearance. They were also correlated with global Aβ and temporal tau SUVR, revealing their impact on Aβ aggregation. The composite score outperformed individual parameters in predicting both Aβ and tau SUVR, suggesting its ability to provide a more comprehensive measure of CSF clearance dysfunction. One could argue that choroidal‐ventricular dysfunction precedes Aβ deposition, as abnormality in our composite score is already present in A−/T− individuals.

Using linear regression voxel wise we found a high association between choroidal‐ventricular parameter composite score and Aβ‐PET and tau‐PET over the cortex, however, the Aβ carried the impact of the composite score on tau. As in normal AD, the Aβ aggregation in extra‐cellular space (where the CSF clearance operates) may contribute to higher accumulation of tau in the neocortex (inside the neurones) ^45^. These findings suggest that impaired CSF clearance not only contributes to Aβ accumulation but indirectly creates an environment conducive to tau deposition and neurodegeneration, further accelerating AD progression.

We found that biomarkers related to Aβ deposition, namely CSF and plasma p‐tau isoforms (181, 217, 231), GFAPs, memory and cognitive scores, were correlated with the choroidal–ventricular parameter composite score, highlighting the superiority of this composite score over other individual parameters, particularly in early‐onset cases. Furthermore, the significant correlation between CSF and plasma Aβ biomarkers with the composite score further supports the role of the CSF clearance system in regulating extracellular waste removal and CSF transition into the bloodstream ^46^.

In line with these cross‐sectional associations, our longitudinal analysis further demonstrated that alterations in the choroidal–ventricular composite score evolve dynamically with AD progression. Specifically, the annualized change in the composite score (ΔComposite/year) increased stepwise across A/T stages in the combined TRIAD + ADNI dataset. This finding supports the notion that choroidal–ventricular dysfunction is not merely a static marker of existing pathology but reflects an active, progressive process that parallels amyloid and tau aggregation over time.

Our findings align with the emerging view that impaired clearance is not merely a downstream consequence of neurodegeneration, but an active factor in AD pathogenesis. Reduced CSF turnover delays the clearance of soluble Aβ and promotes plaque formation, neuroinflammatory processes ^47^, ^48^. The composite score introduced here encompasses multiple aspects of clearance physiology without requiring advanced dynamic imaging techniques. Its early deviation from normative values makes it potentially useful to identify individuals at risk for prevention trials, especially prevention trials of glymphatic or CSF‐mediated clearance mechanisms.

Disturbances in CSF and glymphatic clearance extend beyond Alzheimer's disease to diverse neurological and psychiatric disorders. Impaired brain fluid drainage after traumatic brain injury^49^, choroid plexus enlargement and barrier dysfunction in major depressive disorder^50^, ^51^, and reduced glymphatic flow in Parkinson's disease^52^ collectively implicate the blood–CSF–brain interface^53^ as a shared pathway linking inflammation, impaired clearance, and neurodegeneration across brain disorders.

From a therapeutic perspective, interventions aimed at restoring CSF dynamics (whether through pharmacologic modulation of choroid plexus function, enhancement of glymphatic flow, or devices promoting CSF circulation) could benefit from incorporating this composite metric as a monitoring tool. Moreover, this biomarker may improve patient stratification in clinical trials by identifying subgroups most likely to benefit from clearance‐based interventions.

## 5 LIMITATIONS AND FUTURE DIRECTIONS

Future studies should supplement our results in several ways. First, while ventricular radioactivity is an intermediate of CSF flow, it does not capture real‐time clearance kinetics. More sophisticated imaging modalities such as dynamic PET‐ or MRI‐based CSF flow mapping may supplement our approach. Second, the TRIAD and ADNI cohorts, though large and well‐characterized, do not fully represent global population diversity, warranting validation in multi‐ethnic cohorts. Third, our exclusion of individuals with major comorbidities limits generalizability to real‐world clinical populations, where mixed pathologies are common.

Future research should also address mechanistic questions: How do vascular health, glymphatic integrity, and sleep disturbances (factors known to affect clearance) interact with these ventricular parameters? Can interventions be targeting systemic clearance (e.g., via peripheral sinks or anti‐inflammatory strategies) influence composite score trajectories? Answers to these questions could broaden the role of clearance‐based biomarkers in personalized treatment strategies. Another limitation is that choroid plexus segmentation was performed using FreeSurfer, which may show intersubject variability. In future studies, we will adopt deep‐learning–based segmentation methods that provide greater precision and reproducibility, potentially improving sensitivity^54^.

## 6 CONCLUSION

The choroidal–ventricular segments and composite score introduced here are associated with early and late disease pathophysiology, bridging structural and functional imaging measures. By identifying abnormalities that precede hallmark protein aggregation and clinical symptoms, one provides a promising tool for early detection, mechanistic research, and therapeutic monitoring in AD.

## CONFLICT OF INTEREST STATEMENT

Henrik Zetterberg has served at scientific advisory boards and/or as a consultant for Abbvie, Acumen, Alector, Alzinova, ALZpath, Amylyx, Annexon, Apellis, Artery Therapeutics, AZTherapies, Cognito Therapeutics, CogRx, Denali, Eisai, Enigma, LabCorp, Merck Sharp & Dohme, Merry Life, Nervgen, Novo Nordisk, Optoceutics, Passage Bio, Pinteon Therapeutics, Prothena, Quanterix, Red Abbey Labs, reMYND, Roche, Samumed, ScandiBio Therapeutics AB, Siemens Healthineers, Triplet Therapeutics, and Wave. He has given lectures sponsored by Alzecure, BioArctic, Biogen, Cellectricon, Fujirebio, LabCorp, Lilly, Novo Nordisk, Oy Medix Biochemica AB, Roche, and WebMD. He is a co‐founder of Brain Biomarker Solutions in Gothenburg AB (BBS), which is part of the GU Ventures Incubator Program, and a shareholder of CERimmune Therapeutics (all outside the submitted work). Marcel S. Woo receives honoraria from Lilly and Eisai outside the scope of this manuscript. Karine Provost serves as consultant for Eli Lilly, Biospective, and Optina Diagnostics. All other authors declare no competing interests.

## CONSENT STATEMENT

All participants provided written informed consent.

## Supporting information



Supporting Information

Supporting Information

Supporting Information

Supporting Information

Supporting Information

## Data Availability

All requests for raw and analyzed data and materials from the TRIAD cohort will be promptly reviewed by McGill University to verify if the request is subject to any intellectual property or confidentiality obligations. Anonymized data will be shared upon request to the study's senior author from a qualified academic investigator for the sole purpose of replicating the procedures and results presented in this article. Any data and materials that can be shared will be released via a material transfer agreement. Data are not publicly available due to information that could compromise the privacy of research participants. Related documents, including study protocol and informed consent forms, can similarly be made available upon request.
