# Supplementary material for: Choroidal–ventricular system abnormalities are linked to amyloid‐β aggregation in Alzheimer's disease
Source: Alzheimers Dement. 2026 Feb 25;22(2):e71205. doi: 10.1002/alz.71205 (PMC12933412; doi:10.1002/alz.71205)
Supplement: Supplementary file 1 — Supporting Information [file ALZ-22-e71205-s004.docx]

Supplemental Table 1. The full demographics of participant in TRIAD cohort.

| **Characteristic** | **CU (Y)** | **A-T-** | **A+T-** | **A-T+** | **A+T+** |
| --- | --- | --- | --- | --- | --- |
| **Number** | 51 | 166 | 64 | 5 | 99 |
| **Mean age, years (SD)** | 22.43 (1.75) | 67.77 (9.54) | 70.45 (17.34) | 67.2 ( 17.42) | 79.04 (17.42) |
| **Female, number (%)** | 31 (60.78) | 104 (62.65) | 38 (59.37) | 3 (60.00) | 81 (81.81) |
| **Mean education, years (SD)** | 16.70 (1.76) | 15.86 (3.51) | 15.28 (3.51) | 16.04 (3.51) | 14.84 (3.49) |
| **Mean MMSE score, (SD)** | 29.78 (0.32) | 29.18 (0.82) | 28.24 (3.35) | 22.50 (3.36) | 22.53 (4.29) |
| **Mean CDR scores, (SD)** | 0 (0) | 0 (0) | 0.24 (0.36) | 0.87 (0.36) | 0.83 (0.46) |
| **Mean CDR SOB scores, (SD)** | 0 (0) | 0.04 (1.39) | 0.84 (1.55) | 3.62 (3.03) | 4.12 (3.55) |
| ***APOE* ε4, number (%)** | 11 (21.56) | 36 (25.00) | 23 (35.93) | 1 (25.00) | 53 (53.53) |
| **Plasma GFAP, mean (SD)** | 94.08 (60.89) | 184.11 (90.72) | 284.41 (144.58) | 291.11 (145.00) | 379.67 (147.51) |
| **CSF Aβ 42, mean (SD)** | 783.04 (234.74) | 1114.80 (395.01) | 639.29 (386.21) | 289.5 (386.54) | 498.32 (391.22) |
| **pTau 181, mean (SD)** | 7.61 (3.43) | 9.69 (4.46) | 14.76 (9.20) | 19.31 (9.26) | 22.00 (9.42) |
| **Total amyloid SUVR, mean (SD)** | 1.17 (0.07) | 1.25 (0.10) | 2.04 (0.56) | 1.40 (0.56) | 2.47 (0.61) |
| **Total tau SUVR, mean (SD)** | 0.89 (0.09) | 0.92 (0.09) | 0.99 (65) | 2.84 (0.65) | 2.33 (0.78) |
