# Supplementary material for: Choroidal–ventricular system abnormalities are linked to amyloid‐β aggregation in Alzheimer's disease
Source: Alzheimers Dement. 2026 Feb 25;22(2):e71205. doi: 10.1002/alz.71205 (PMC12933412; doi:10.1002/alz.71205)
Supplement: Supplementary file 2 — Supporting Information [file ALZ-22-e71205-s003.docx]

Supplemental Table2. The full demographics of participant in ADNI cohort.

| **Characteristic** | **A-T-** | **A+T-** | **A-T+** | **A+T+** |
| --- | --- | --- | --- | --- |
| **Number** | 185 | 66 | 2 | 29 |
| **Mean age, years (SD)** | 74.11 (9.01) | 79.90 (7.75) | 71.10 (9.21) | 75.69 (7.67) |
| **Female, number (%)** | 99 (53.51) | 31 (46.96) | 1 (50.00) | 13 (44.82) |
| **Mean education, years (SD)** | 16.42 (2.62) | 16.46 (2.62) | 17.00 (3.00) | 15.13 (2.58) |
| **Mean MMSE score, (SD)** | 29.07 (2.75) | 27.45 (2.78) | 22.5 (0.50) | 23.31 (2.95) |
| **Mean CDR SOB scores, (SD)** | 0.04 (1.84) | 1.34 (1.85) | 3.50 (2.00) | 4.51 (2.12) |
| **APOE ε4, number (%)** | 52 (28.10) | 28 (42.42) | 1 (50.00) | 18 (62.06) |
| **Plasma GFAP, mean (SD)** | 0.01 (0.04) | 0.14 (0.04) | 0.07 (0.00) | 0.15 (0.4) |
| **CSF Aβ 42, mean (SD)** | 1478.33 (727.71) | 794.95 (735.02) | 411.04 (343.44) | 553.42 (661.75) |
| **Total amyloid SUVR, mean (SD)** | 0.93 (0.19) | 1.25 (0.18) | 0.93 (0.08) | 1.34 (0.18) |
| **Total tau SUVR, mean (SD)** | 1.14 (0.19) | 1.18 (0.20) | 1.80 (0.40) | 1.65 (0.20) |
