# Supplementary material for: Choroidal–ventricular system abnormalities are linked to amyloid‐β aggregation in Alzheimer's disease
Source: Alzheimers Dement. 2026 Feb 25;22(2):e71205. doi: 10.1002/alz.71205 (PMC12933412; doi:10.1002/alz.71205)
Supplement: Supplementary file 3 — Supporting Information [file ALZ-22-e71205-s002.docx]

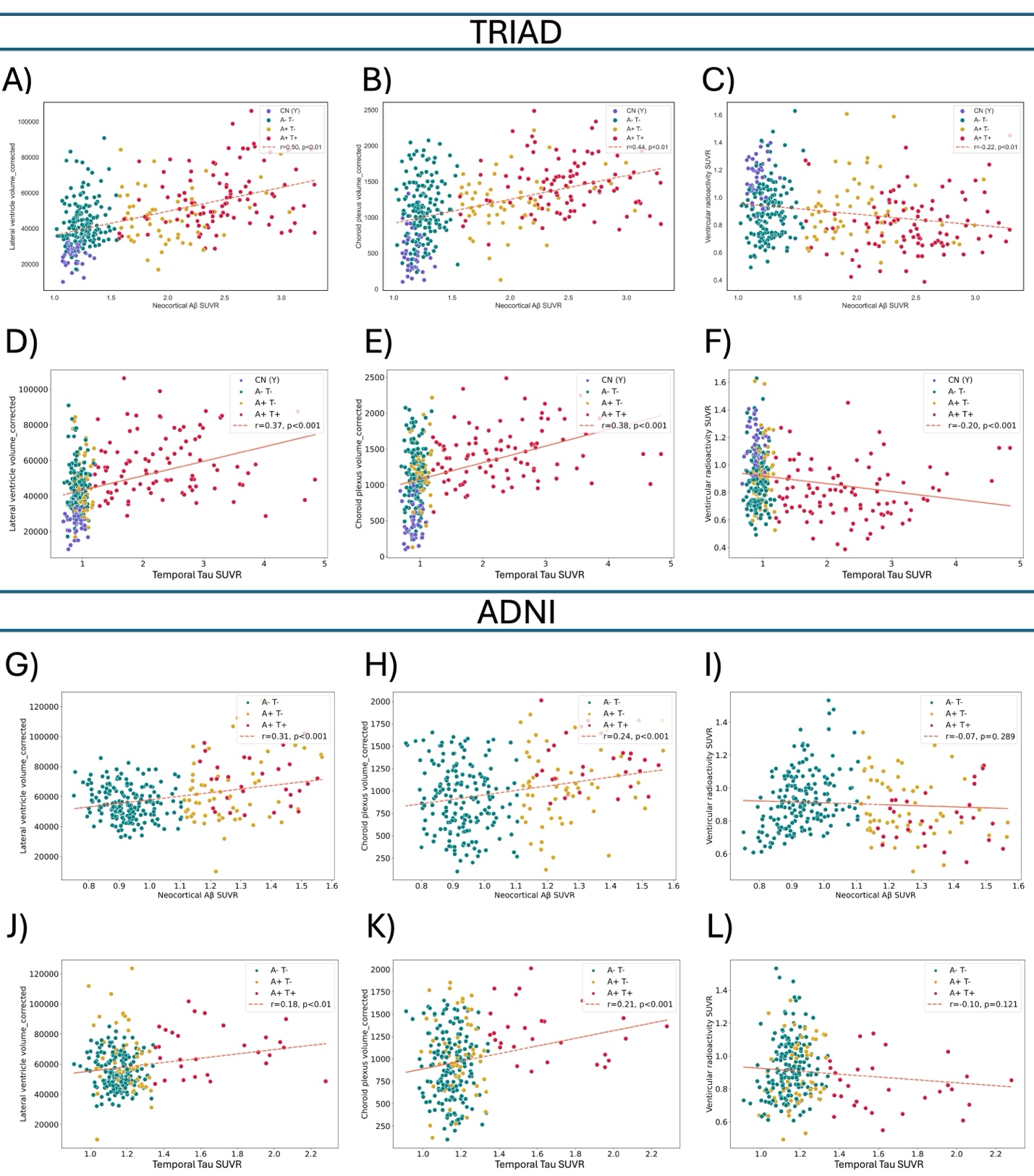


Supplemental Figure 1. Levels of AD hallmark proteins significantly correlate with choroidal-ventricular parameters.
