# Supplementary material for: Choroidal–ventricular system abnormalities are linked to amyloid‐β aggregation in Alzheimer's disease
Source: Alzheimers Dement. 2026 Feb 25;22(2):e71205. doi: 10.1002/alz.71205 (PMC12933412; doi:10.1002/alz.71205)
Supplement: Supplementary file 4 — Supporting Information [file ALZ-22-e71205-s005.docx]

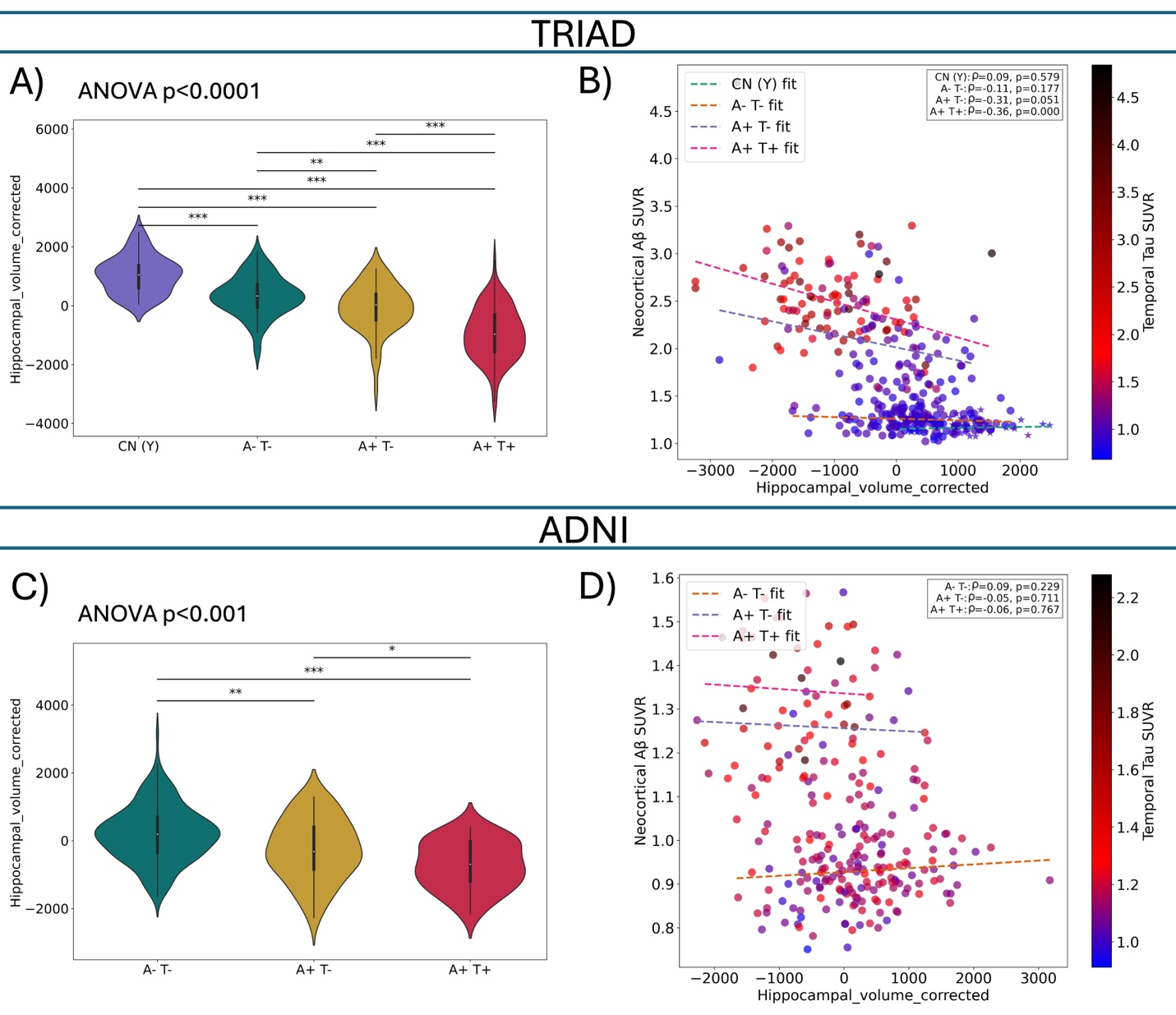
 Supplemental Figure 2. Hippocampal atrophy reflects neurodegeneration but fails to capture early amyloid changes, unlike the choroidal-ventricular parameter composite score.

Legend: Panels show group differences and associations between hippocampal volume (corrected for intracranial volume) and neocortical Aβ SUVR in TRIAD (A, B) and ADNI (C, D). Violin plots demonstrate significant hippocampal atrophy across biomarker-defined groups (ANOVA p < 0.001), consistent with its role as a neurodegeneration marker (A, C). Scatter plots (B, D) reveal weak or absent associations between hippocampal volume and neocortical Aβ, even in A-T- and A+T- individuals, after multiple-comparison correction. Unlike the choroidal-ventricular parameter composite score (Fig. 2), which showed robust associations with Aβ and preceded CSF Aβ42 reduction, hippocampal atrophy did not predict early amyloid accumulation, highlighting its downstream role in disease progression.
